# Supplementary material for: Estimation of country-level basic reproductive ratios for novel Coronavirus (SARS-CoV-2/COVID-19) using synthetic contact matrices
Source: PLoS Comput Biol. 2020 Jul 2;16(7):e1008031. doi: 10.1371/journal.pcbi.1008031 (PMC7363110; doi:10.1371/journal.pcbi.1008031)
Supplement: S1 Appendix — (PDF) [file pcbi.1008031.s001.pdf]

Appendix to *Estimation of country-level basic reproductive ratios for novel Coronavirus (SARS-CoV-2/COVID-19) using synthetic contact matrices*

Joe Hilton[a,b] and Matt J. Keeling[a,b,c]

[a]School of Life Sciences, University of Warwick, Coventry, CV4 7AL, UK
[b]Zeeman Institute (SBIDER), University of Warwick, Coventry, CV4 7AL, UK
[c]Mathematics Institute, University of Warwick, Coventry, CV4 7AL, UK

June 20, 2020

1 Methods

1.1 Model description

We model transmission in a population divided into K discrete age classes $C_1, ...C_K$. Denote by $k_{a,b}$ the expected number of contacts with individuals of age class C_a made per day by a single individual of age class C_b. The matrix $\boldsymbol{K} = (k_{a,b})$ will be asymmetric, with the ath row corresponding to the contacts made per day by an average individual of age class C_a, and the ath column corresponding to an average individual of each age class's contacts with age class C_a. Scaling through by an appropriate β to give $\beta_{a,b}$ will give us an age-structured transmission matrix. We assume that infectious individuals of every age class recover at rate γ. Each age class has an age-specific susceptibility σ_a, which acts by scaling the rate at which individuals in that age class contract infection. Throughout, we make the assumption that there is a close correlation between showing symptoms and being detected, so that we can assume that case data describes all the symptomatic cases to have presented so far, and that any cases not in the data have not shown symptoms. We denote the probability that a case in age class C_a becomes symptomatic and is detected by d_a, and suppose that the rate at which the asymptomatic, undetected cases in age class C_a transmit infection is scaled by a factor $\tau_a \leq 1$. We obtain the following ODE model, where S_a is the number of susceptible individuals, E_a the number of exposed individuals, D_a the number of symptomatic cases which are currently infectious, and U_a the number of asymptomatic currently infectious cases in age class C_a:

$$\begin{aligned}
\frac{\mathrm{d}S_a}{\mathrm{d}t} &= -\sigma_a \sum_b \beta_{a,b}[D_b + \tau_b U b] \\
\frac{\mathrm{d}E_a}{\mathrm{d}t} &= \sigma_a \sum_b \beta_{a,b}[D_b + \tau_b U b] - \alpha E_a \\
\frac{\mathrm{d}D_a}{\mathrm{d}t} &= \alpha d_a E_a - \gamma D_a \\
\frac{\mathrm{d}U_a}{\mathrm{d}t} &= \alpha(1 - d_a)E_a - \gamma U_a.
\end{aligned}$$

The early dynamics of this system can be captured in a next-generation matrix. Setting $r_0 = \beta/\gamma$, the matrix $r_0\boldsymbol{K}$ captures the total expected number of age-structured infectious contacts made over an individual's entire infectious period. The relationship between the age-stratified cases in generation $t+1$, D_a^{t+1}, and those in generation t, D_a^t, is given by

$$\begin{aligned}
D_a^{t+1} &= d_a \sum_b \sigma_a r_0 k_{a,b}[D_b^t + \tau_b U_b^t] \\
&= d_a \sigma_a \sum_b r_0 k_{a,b}[1 + \tau_b \frac{1 - d_b}{d_b}]D_b^t \\
&= \sum_b [d_a \sigma_a r_0 k_{a,b} T_b] D_b^t,
\end{aligned}$$

where $T_b = (1 + \tau_b \frac{1-d_b}{d_b})$, and we have used the fact that $D_b^t = \frac{1-d_b}{d_b} U_b^t$. In practice, we do not particularly care what units σ_a is expressed in, we can just rescale the entire vector $\underline{\sigma}$ by this scaling factor. This gives us a next generation matrix $\widehat{\boldsymbol{R}} = [d_a \sigma_a k_{a,b} T_b]$, so that

$$\underline{D}_{t+1} = \underline{D}_t \widehat{\boldsymbol{R}},$$

where $\underline{D}_t$ is a vector with ath entry D_a^t. In the early growth phase of the epidemic, the leading eigenvalue of this matrix is the basic reproductive ratio R_0, and the corresponding eigenvector gives the age-stratified distribution of symptomatic cases[1]. This is captured by the equation

$$\underline{D}\widehat{\boldsymbol{R}} = R_0 \underline{D},$$

or

$$d_a \sigma_a \sum_b k_{a,b} D_b T_b = R_0 D_a.$$

The basic reproductive ratio is specifically that of the symptomatic cases, the ratio of the number of symptomatic cases in generation $t+1$ compared to the number in generation t. In general, this will not be the same as the "true" basic reproductive ratio describing the evolution of all infectious cases, but since we are assuming that the reported cases are precisely those which present with symptoms we can interpret it as the ratio we would infer given the reported cases and no consideration of undetected cases.

Using estimates from the literature of the age-stratified distribution of symptomatic cases $\underline{D}$ and the basic reproductive ratio R_0, we can attempt to infer the age-stratified susceptibility, symptomatic, and asymptomatic transmissibility profiles $\underline{\sigma}$, $\underline{d}$, and $\underline{\tau}$. This would involve inferring K parameters from just $K+1$ data points (the K age-stratified proportions plus the basic reproductive ratio), and so we will consider two specific cases where fitting is possible.

1.2 Homogeneous $\sigma_a \equiv \sigma$, homogeneous $d_a \equiv d$, homogeneous $\tau_a \equiv \tau$

The first case we consider corresponds to a setting where age structured transmission is entirely driven by contact behaviour. In this case, all age classes are equally susceptible and show symptoms with probability d, and asymptomatic cases transmit at a rate reduce by the age-independent factor τ. In this case, the next generation relationship is given by

$$D_a^{t+1} = d\sigma \sum_b k_{a,b} [1 + \tau \frac{1-d}{d}] D_a^t.$$

If we introduce $\rho = d\sigma[1 + \tau\frac{1-d}{d}]$ then the next-generation matrix is precisely $\rho \boldsymbol{K}$. In this setting, we fit the single parameter ρ so that our model matches the estimated basic reproductive ratio, ignoring the information we have about the age distribution of cases. We require that the leading eigenvalue of $\widehat{\boldsymbol{R}} = \rho \boldsymbol{K}$ be the estimated R_0, and so the appropriate value is $\rho = fracR_0\lambda$, where λ is the leading eigenvalue of $\boldsymbol{K}$.

1.3 No asymptomatic transmission: $\tau_a \equiv 0$

The second case we consider allows for heterogeneity in susceptibility and symptomatic probability, but assumes transmission from aysmptomatic cases is negligible. In this case, the next generation relationship is given by

$$D_a^{t+1} = d_a \sigma_a \sum_b k_{a,b} D_a^t.$$

We can not independently fit the two vectors $\underline{\sigma}$ and $\underline{d}$, but if we introduce $\rho_a = \sigma_a d_a$, then the single K-dimensional vector $\underline{\rho}$ is sufficient to define the age-structured mixing as follows. The next generation relationship is

$$D_a^{t+1} = \rho_a \sum_b k_{a,b} D_a^t,$$

and the eigenvector equation is then

$$\rho_a \sum_b k_{a,b} D_b = R_0 D_a.$$

We can thus estimate ρ_a as

$$\rho_a = R_0 \frac{D_a}{\sum_b k_{a,b} D_b}.$$

This case includes the two extreme subcases of homogeneous susceptibility with age-specific symptomatic probability ($\rho = \sigma d_a$) and age-specific susceptibility with homogeneous symptomatic probability ($\rho = \sigma_a d$), along with a continuum of intermediate cases where both factors contribute to age-structured heterogeneity.

In the specific data we are using in this study, our case data is stratified into nine age classes corresponding to the eight ten-year intervals from 0 to 80, plus a class of individuals aged 80+[2], while the contact frequency matrices are stratified according to sixteen age classes corresponding to the fifteen five-year intervals from 0 to 75 plus a class of individuals aged 75+[3]. To carry out the fitting procedure we therefore need to convert both the case data and the contact matrix for China so that they are both expressed in terms of the same age structure. The finest age structure which both can be reduced to consists of the eight ten-year intervals from 0 to 70, plus a class of individuals aged 70+. Converting the data is easy: we just combine the 70-80 and 80+ classes by adding together the number of cases in both. To convert the contact matrix, denote the contact matrix in terms of five-year intervals by $\underline{K}$ and the matrix in terms of ten-year intervals by $\underline{\tilde{K}}$, and consider the two adjacent five-year age classes C_{2a-1} and C_{2a} which together make up the single ten-year age class $\tilde{C}_a$. Then, denoting the number of individuals in a given age class C by $|C|$, the conversion is given by

$$\tilde{k}_{a,b} = \frac{1}{|C_{2a-1}| + |C_{2a}|}[|C_{2a-1}|(k_{2a-1,2b-1} + k_{2a-1,2b}) + |C_{2a}|(k_{2a,2b-1} + k_{2a,2b})],$$

so that the expected contacts made by an individual in class $\tilde{C}_a$ with individuals in class $\tilde{C}_b$ are the weighted average of the total expected contacts made by individuals in the two finer age classes which make up class $\tilde{C}_a$ with individuals from the two finer age classes that make up class class $\tilde{C}_b$.

The estimated values of ρ_a are as follows:

Age class	ρ_a
0 to 10	0.007720
10 to 20	0.006734
20 to 30	0.035369
30 to 40	0.064782
40 to 50	0.075954
50 to 60	0.155723
60 to 70	0.376402
70+	0.520127

These estimates demonstrate that older people are substantially more likely to become infected and develop symptoms as a result of contact with an infectious individual than younger people, in contrast with the purely contact frequency-based approach where all age classes are equally likely to develop symptoms after a contact event. We can make this profile consistent with the finer five-year age intervals used by Prem *et al.* by simply assigning ρ_a to any of the constituent finer age classes that might make up the coarser age class $\tilde{C}_a$, and so the contact matrix for China is the only one that ever needs to be converted to a coarser age structure.

1.4 Calculation of country-specific basic reproductive ratio

To estimate the next generation matrix $\widehat{\boldsymbol{R}}$ for some new population with age-structured contact matrix $\tilde{\boldsymbol{k}} = (\tilde{k}_{i,j})$, we assume that the age-specific susceptibility, symptomatic probability, and asymptomatic transmissibility profiles are the same for all populations. Then the next-generation mapping for age class C_a in the new population is given by

$$D_a^{t+1} = \sum_b [d_a \sigma_a \tilde{k}_{a,b} T_b] D_b^t,$$

and the eigenvalue of the matrix $[d_a \sigma_a \tilde{k}_{a,b} T_b]$ gives us the estimated basic reproductive ratio $\tilde{R}_0$ for the new population. Notice that in both the special cases we considered, the susceptibility profile $\underline{\sigma}$ was linear in R_0, and so the next generation matrix for our new population will also be linear in the R_0 estimate from the original population. This means that given the R_0 estimate for our original population, the basic reproductive ratios for every other country are just linear scalings of this estimate, with a different scaling factor for each country. In a practical setting, we can calculate the appropriate scaling factors by setting

the estimated R_0 to 1, and then store these scaling factors in a table which we can refer to whenever a new estimate of R_0 in China becomes available.

2 Tables of basic reproductive ratio by country

Country	R_0 (null)	R_0 (China CDC)	Country	R_0 (null)	R_0 (China CDC)
Albania	2.64	2.27	Ecuador	2.79	1.87
Algeria	2.87	1.36	Egypt	3.17	1.43
Andorra	2.06	2.37	El Salvador	3.10	1.81
Antigua and Barbuda	2.74	2.16	Estonia	2.02	3.26
Argentina	2.33	2.11	Ethiopia	4.02	2.06
Armenia	2.45	2.46	Fiji	2.81	1.65
Australia	2.20	2.56	Finland	1.97	3.64
Austria	2.29	3.01	France	2.01	2.70
Azerbaijan	2.92	2.16	Georgia	2.25	2.85
The Bahamas	2.92	2.24	Germany	1.22	1.99
Bahrain	2.67	2.34	Ghana	3.01	2.04
Bangladesh	3.09	1.62	Greece	1.99	3.14
Belarus	2.14	2.59	Guatemala	3.58	1.46
Belgium	1.95	3.60	Guinea	3.63	1.89
Belize	3.48	1.70	Guyana	3.12	1.69
Benin	3.85	2.08	Haiti	2.97	1.99
Bhutan	2.84	2.05	Honduras	3.22	1.66
Bolivia	2.96	2.01	Hong Kong SAR, China	2.29	2.75
Bosnia and Herzegovina	2.39	2.77	Hungary	1.97	3.09
Botswana	3.14	1.67	Iceland	2.38	2.68
Brazil	2.79	2.00	India	2.78	1.73
Brunei	2.69	1.84	Indonesia	2.68	2.02
Bulgaria	1.92	3.52	Iran	2.59	1.53
Burkina Faso	3.78	1.75	Iraq	3.61	1.79
Cabo Verde	3.80	1.37	Ireland	2.13	2.27
Cambodia	3.70	2.14	Israel	2.18	2.09
Cameroon	3.59	2.05	Italy	2.44	4.18
Canada	2.17	2.76	Jamaica	2.81	1.91
Chile	2.32	2.25	Japan	1.89	4.13
China	2.40	2.44	Jordan	3.18	1.21
Colombia	2.68	1.99	Kazakhstan	2.80	2.26
Republic of the Congo	2.99	1.65	Kenya	3.32	1.66
Costa Rica	2.68	1.96	Kiribati	2.94	1.47
Croatia	1.95	3.15	Kuwait	2.73	2.30
Cyprus	2.24	2.58	Kyrgyzstan	2.97	1.74
Czech Republic	2.14	3.19	Laos	3.51	1.60
Denmark	2.14	3.18	Latvia	2.12	3.13
Dominican Republic	2.70	1.85	Lebanon	2.45	1.72

Table A: Estimated basic reproductive ratio by country under the null purely contact-structured model and under the assmption of further age-specific heterogeneities fitted to the China CDC data, page one of two.

Country	R_0 (null)	R_0 (China CDC)	Country	R_0 (null)	R_0 (China CDC)
Lesotho	3.31	1.82	Saudi Arabia	2.67	1.74
Liberia	3.34	1.64	Senegal	3.58	1.45
Lithuania	2.33	2.73	Serbia	1.93	3.36
Luxembourg	2.82	3.79	Seychelles	2.37	2.15
Malaysia	2.56	2.00	Sierra Leone	3.59	1.93
Maldives	4.13	1.73	Singapore	2.34	2.68
Malta	2.26	3.08	Slovakia	2.30	2.68
Mauritania	3.50	1.39	Slovenia	2.01	2.90
Mauritius	2.15	2.10	Solomon Islands	3.35	1.53
Mexico	2.65	1.80	South Africa	2.66	1.76
Monaco	1.79	6.95	Spain	2.00	2.79
Mongolia	2.65	1.79	Sri Lanka	2.72	1.75
Montenegro	2.20	2.48	Suriname	2.35	1.95
Morocco	2.64	1.66	Sweden	2.14	3.21
Mozambique	3.93	1.88	Switzerland	2.34	3.05
Namibia	3.22	1.96	Syria	3.09	1.32
Nepal	3.53	2.18	Taiwan	2.31	2.32
Netherlands	2.60	3.44	Tajikistan	3.69	1.58
New Zealand	2.26	2.71	Republic of Macedonia	2.35	2.47
Nicaragua	3.27	1.64	Thailand	2.36	2.54
Niger	4.55	1.50	East Timor	3.68	1.39
Nigeria	3.46	1.69	Tonga	3.38	1.27
Oman	2.69	1.97	Tunisia	2.72	1.57
Pakistan	3.75	1.36	Turkey	2.50	1.71
Panama	2.47	1.82	Uganda	4.18	2.04
Paraguay	2.95	1.89	Ukraine	2.15	3.04
Peru	2.75	2.13	United Arab Emirates	3.08	2.62
Philippines	3.02	1.85	United Kingdom	1.67	2.04
Poland	2.45	3.99	Tanzania	3.74	2.10
Portugal	1.99	3.11	United States of America	2.22	2.47
Qatar	3.37	3.13	Uruguay	2.30	2.41
South Korea	2.34	2.66	Uzbekistan	3.92	1.22
Romania	1.98	2.70	Vanuatu	3.16	1.75
Russia	2.18	2.72	Venezuela	2.43	1.96
Rwanda	3.21	2.06	Vietnam	2.61	2.41
Saint Lucia	2.99	1.89	Yemen	3.77	1.06
Samoa	2.94	1.08	Zambia	3.75	1.91
Sao Tome and Principe	3.13	1.00	Zimbabwe	3.65	1.72

Table B: Estimated basic reproductive ratio by country under the null purely contact-structured model and under the assmption of further age-specific heterogeneities fitted to the China CDC data, page two of two.

References

[1] Keeling MJ, Rohani P. Modeling infectious diseases in humans and animals. Princeton University Press; 2011.

[2] Novel Coronavirus Pneumonia Emergency Response Epidemiology Team. The epidemiological characteristics of an outbreak of 2019 novel coronavirus diseases (COVID-19) in China. China CDC Weekly. 2020;41(2):145.

[3] Prem K, Cook AR, Jit M. Projecting social contact matrices in 152 countries using contact surveys and demographic data. PLoS computational biology. 2017;13(9):e1005697.
