# Supplementary material for: Estimation of country-level basic reproductive ratios for novel Coronavirus (SARS-CoV-2/COVID-19) using synthetic contact matrices
Source: PLoS Comput Biol. 2020 Jul 2;16(7):e1008031. doi: 10.1371/journal.pcbi.1008031 (PMC7363110; doi:10.1371/journal.pcbi.1008031)
Supplement: Attachment — Submitted filename: PCOMPBIOL-D-20-00579.pdf [file pcbi.1008031.s003.pdf]

PCOMPBIOL-D-20-00579

The authors have well described the motivation for their study and cited relevant literature both from the historic approaches to pandemics/epidemics and from the current SARS-CoV-2/COVID-19 pandemic.

I am satisfied with the model as written and the assumptions of the model are well described in the Appendix, particularly the implications of the assumptions regarding the difference between the "true" R_0 and that which would be estimated under assumptions of under-reporting.

The article provides useful information to the epidemiology community regarding expected differences in transmission in the absence of interventions, indicating that assumptions of age-structured effects play a role in explaining differences from one country to another.

The modelling is of high quality and my comments below focus on the interpretability of the figures and tables which I feel let down the high quality modelling. Other than this I am very happy with the article and would recommend it for publication after the figures and tables are amended.

Results

Line 135: "If contrast" should be "In contrast"

Line 154: "probably of symptoms" should be "probability of symptoms"

Figure 1: The authors discuss green countries on the map as having a lower R_0 than China and the red having R_0 higher than China's. The authors do not describe what a blue shade represents. Further, the blue shades are difficult to distinguish between, and the boundaries between greens and blues and then blues and reds is different across subplots (a) and (b), likely as a result of the maximum and minimum R_0 values being different across the plots. Additionally, there does not appear to be a strict linear spacing in the colourbars in the legend. This figure should be rebuilt with an obvious perceptual boundary at China's R_0 value representing the transition the authors describe, e.g. choosing only two colours and having a gradient through a neutral tone. `#FF0000` and `#00FF00` are poor choices of opposing colour given the prevalence of colourblindness in the population. The authors should ensure that this new colour gradient is identical across (a) and (b) to enable easy visual comparison, that the colourbar has a linear scale, and that the distance from China's R_0 in either direction has equal visual weight. The authors could additionally gain a little extra contrast by clipping the upper limit of the colour scale at 4 as only a handful of countries have an R_0 higher than this under either model.

Figure 2: The authors should mark China in this figure given its importance in both the pandemic and parameter estimation. Another country of interest in the discussion around the differences between the two models is Germany, whose null model R_0 is the smallest at 1.22. Under heterogeneous susceptibility assumptions the R_0 value is 1.99, and this is nearly as an extreme ratio as Italy, and indicates that under assumptions of homogeneous susceptibility Germany would assume to be better placed to control the outbreak than may be warranted. I imagine for

similar reasons of leaving Monaco out of this analysis that Sao Tome and Principe and Samoa have been left out as they have an R_0 only slightly greater than 1 under the heterogeneous model, $R_0 \approx 3$ under the null model, and small populations.

Appendix

The tables in Section 2 are laid out in a way which makes it difficult to compare the estimates under the competing assumptions. Could the authors please round their estimates to two decimal places and present these side by side for each country? I found myself wanting to compare which countries had large disparities in estimated R_0 and the simplest way to do this was to look on the scatter plot in Figure 2 and then hunt across the two Appendix tables, one at a time, to determine likely candidates for the point I was interested in.
