# Supplementary material for: Estimation of country-level basic reproductive ratios for novel Coronavirus (SARS-CoV-2/COVID-19) using synthetic contact matrices
Source: PLoS Comput Biol. 2020 Jul 2;16(7):e1008031. doi: 10.1371/journal.pcbi.1008031 (PMC7363110; doi:10.1371/journal.pcbi.1008031)
Supplement: Attachment — Submitted filename: Hilton-Keeling-Response-to-Reviewers.docx [file pcbi.1008031.s004.docx]

University of Warwick

Friday 29^th^ May 2020

Dear Editors,

We would like to thank yourselves and the reviewers for their insightful comments and corrections and for taking the time to process and review our manuscript. In line with the reviewer’s comments, we have made the following revisions:

- the colour palette on the maps in Figure 1 now increments from blue (low $R_{0}$) through yellow (intermediate) to red (high), resulting in what we feel is a more readable pair of maps. Our palette was chosen from a selection of colour-blindness-friendly palettes;
- the values of $R_{0}$ estimated for China and Germany have been identified on the scatter plot in Figure 2(a), with a sentence in the caption mentioning the substantial proportional difference between the two estimates for Germany which was identified by Reviewer 2;
- the tables of estimated $R_{0}$ values at the end of the Appendix have been combined into a single table over two pages which include both sets of estimates;
- corrections to various typos.

In addition to the revisions recommended by the reviewers, we have also slightly updated the first paragraph of our introduction to reflect the continued progress of the pandemic since our original draft. In the “tracked” version of our manuscript we have identified our revisions by highlighting deleted sections of the text in red, and insertions in green. We have also updated the Github repository to include our calculated $R_{0}$ estimates as well as a function scaling_to_R0.m which calculates these estimates from a set of scaling factors based on an $R_{0}$ for China.

We once again thank the reviewers for their helpful comments, and look forward to submitting to *PLOS Computational Biology* again in future.

Yours

Joe Hilton & Matt Keeling
